# Mathematical models of malaria - a review

**DOI:** 10.1186/1475-2875-10-202

**Published:** 2011-07-21

**Authors:** Sandip Mandal, Ram Rup Sarkar, Somdatta Sinha

**Affiliations:** 1 Centre for Cellular and Molecular Biology (CSIR), Uppal Road, Hyderabad 500007, India

## Abstract

Mathematical models have been used to provide an explicit framework for understanding malaria transmission dynamics in human population for over 100 years. With the disease still thriving and threatening to be a major source of death and disability due to changed environmental and socio-economic conditions, it is necessary to make a critical assessment of the existing models, and study their evolution and efficacy in describing the host-parasite biology. In this article, starting from the basic Ross model, the key mathematical models and their underlying features, based on their specific contributions in the understanding of spread and transmission of malaria have been discussed. The first aim of this article is to develop, starting from the basic models, a hierarchical structure of a range of deterministic models of different levels of complexity. The second objective is to elaborate, using some of the representative mathematical models, the evolution of modelling strategies to describe malaria incidence by including the critical features of host-vector-parasite interactions. Emphasis is more on the evolution of the deterministic differential equation based epidemiological compartment models with a brief discussion on data based statistical models. In this comprehensive survey, the approach has been to summarize the modelling activity in this area so that it helps reach a wider range of researchers working on epidemiology, transmission, and other aspects of malaria. This may facilitate the mathematicians to further develop suitable models in this direction relevant to the present scenario, and help the biologists and public health personnel to adopt better understanding of the modelling strategies to control the disease

## Background

Malaria is an ancient disease having a huge social, economic, and health burden. It is predominantly present in the tropical countries. Even though the disease has been investigated for hundreds of years, it still remains a major public health problem with 109 countries declared as endemic to the disease in 2008. There were 243 million malaria cases reported, and nearly a million deaths - primarily of children under 5 years [1]. With no effective vaccine in sight and many of the older anti-malarial drugs losing effectiveness due to the parasite evolving drug resistance, prevention (using bed nets) is still the only advisory given to afflicted persons. Malaria has also gained prominence in recent times since climate change or global warming is predicted to have unexpected effects on its incidence. Both increase and fluctuation in temperature affects the vector and parasite life cycle. This can cause reduced prevalence of the disease in some areas, while it may increase in others. Thus climate change can affect malaria prevalence pattern by moving away from lower latitudes to regions where populations have not developed immunity to the disease [2-8].

Malaria is caused by the protozoan parasites of genus *Plasmodium*. In humans it is caused by *Plasmodium falciparum, Plasmodium malariae, Plasmodium ovale*, and *Plasmodium vivax*. Of these, *P. falciparum *is the most common cause of infection in Africa and South East Asia, and is responsible for ~80% of all malaria cases and ~90% of deaths [1]. In India, *P. vivax*, has been the primary pathogen responsible for malaria, even though *P. falciparum *cases are on the rise in recent times [9]. The parasite requires two hosts to complete its life cycle - the vector female *Anopheles *mosquito and human. The bites/bloodmeals of infected mosquitoes are the mode of transmission of the parasite between the human hosts. Grassi and Ross discovered the mosquito's role in the parasite life cycle and transmission in 1897 [1], and the genomes of *Anopheles *mosquito and *P. falciparum *were sequenced in 2002 [10,11]. During the interim 105 years, much scientific research was undertaken and progress made in the understanding of the host-parasite-vector interactions and their biology. However, the complexities in the life cycle of the parasite, highly complex environmental and social interactions, evolutionary pressure of drugs and control measures contributing to drug resistance of parasite, unforeseen effects of climate change, and migration of population between endemic and non endemic areas continued to contribute to the huge burden of morbidity and mortality accompanying the disease. These have also thrown up new challenges to researchers and public health professionals.

Among all areas in Biology, researchers in infectious disease were one of the foremost to realize the important role of mathematics and mathematical models in providing an explicit framework for understanding the disease transmission dynamics within and between hosts and parasites. In a mathematical expression or a model, several known clinical and biological information are included in a simplified form by selecting features that seem to be important to the question being investigated in disease progression and dynamics. Therefore, a model is an "approximation" of the complex reality, and its structure depends upon the processes being studied and aimed for extrapolation. Based on the questions being asked, these studies can help fit empirical observations, and can be applied to make theoretical predictions on lesser known or unknown situations. For example, mathematical models have been widely used by epidemiologists as tools to predict the occurrence of epidemics of infectious diseases, and also as a tool for guiding research for eradication of malaria at the present time [12,13].

Malaria is one of the oldest diseases studied for a long time from all angles, and vast literature exists describing a host of modelling approaches. Different approaches are helpful in guiding different stages of the disease through synthesizing available information and extrapolating it. It is felt that a combination of different approaches, rather than a single type of modelling, may have long term usefulness in eradication and control [13]. In the recent years, global eradication and control efforts [14,15] have led to a surge of activities leading to many studies and publications. It is a formidable task to review all types of models in one article. In this article a historical path has been considered, and an attempt is made to take into account some of those mathematical models, which are primarily focused on the transmission dynamics of the infection in the host and vector populations, using the epidemiological compartment modelling approach [16,17]. The modelling methodology is predominantly deterministic and differential equation based.

This in no way undermines the importance of other models that are concerned with the "within host" biology, or population genetic models that have an increasing impact in eradication and control. To study the infection phenomena inside the individual host, "within host" models consider the interaction of the parasite with the immune cells in an individual host [18-21]. Population genetic models study evolution and spread of the parasite in a complex landscape of varying host immunity, host death, drugs, and mosquito availability [22,23]. These are connected to the parasitological status of a population, which is related to the different classes in the epidemiological compartment models. As mentioned earlier, only few recent papers are referred on these topics, and interested readers may get further leads from them. Different modelling methodologies have also been adopted in addition to differential equation-based models. Few examples are, individual-based models [24], habitat-based models [25], integrated models [26,27], and others [17,28-34]. In spite of the wide range of these models and methodologies, the major modelling approach still remains the transmission of infection through the epidemiological compartments of human and vector populations. Further, with the recent concern with climate change [5,6], the importance of the power of prediction of mathematical models in understanding the infectious disease transmission, highlights the requirement of a consolidated review on this modelling strategy and evolution of the models employed till date.

Sir Ronald Ross, while working at the Indian Medical Service in 1890's, demonstrated the life-cycle of the malaria parasite in mosquito, and was one among the first to publish a series of papers using mathematical functions to study transmission of Malaria in early 1900 [35-39]. He developed a simple model, now known as the classical "Ross model" [36], which explained the relationship between the number of mosquitoes and incidence of malaria in humans. A commonly adopted method of parsimony in developing mathematical models is to accept the simplest possible theoretical description consistent with the data available at a given time. However, these simple models often have limited predictability and are not satisfactory when new data becomes available, and more complexities of interactions are considered. Therefore, subsequently several models have been developed by researchers who extended Ross's model by considering different factors, such as latent period of infection (Table 1) in mosquitoes and human [12,40], age-related differential susceptibility to malaria in human population [12,41,42], acquired immunity [41,43,44], and spatial and genetic heterogeneity of host and parasite [45-49].

**Table 1:** Glossary of different important terms

**Latent period (τ):**	The period from the point of infection to the beginning of the state of infectiousness is known as Latent period during which the infected individuals stay in the exposed (*E*) class.
**Incubation period:**	The period from the point of infection to the appearance of symptoms of disease is known as the Incubation period.
**Asymptomatic:**	In some infections, symptoms do not appear in the individual in spite of being a carrier for a disease and this is called Asymptomatic Infection. The appearance of symptoms is important for case diagnosis and treatment. Sometimes asymptomatic infections are also called subclinical infections.
**Vectorial capacity:**	All the information (vector density relative to host, biting rate, life expectancy etc.) about the vector populations is incorporated through vectorial capacity, which is defined as the number of potentially infective contacts an individual person makes, through the vector population, per unit time.
**Entomological Inoculation Rate (EIR):**	Rate of infectious bites per person is termed as Entomological inoculation rate.
**Force of infection:**	Per capita rate of acquisition of infection by infectious bites is called force of infection.
**Clinical immunity:**	The immunity, which reduces the probability of clinical disease, is called Clinical immunity.
**Anti-parasite immunity:**	The immunity, which is responsible for clearance of parasite is called Anti-parasite immunity.
**Effectiveness of treatment (∈):**	The ratio of the duration of infection for the untreated and treated sensitive parasites.
**Cost of resistance (Γ):**	The reduction of a resistant parasite's fitness relative to that of a sensitive parasite, when neither parasite is exposed to the drug.

With all these models at hand, it is not a trivial matter to infer the crucial features of the disease, and get a coherent understanding of the development of the models from interactions among the vector, parasite and host. In this review, the emphasis is more on the evolution of different mathematical models (mainly differential equation based) of malaria with a brief discussion on stochastic models and data based statistical models. The first aim of this paper is to develop a hierarchical structure of the range of deterministic models of different levels of complexity, starting from the basic Ross Model. The second objective is to elaborate on the evolution of modelling strategies in different steps, using some of the key mathematical models that describe malaria incidence by including specific properties of host-vector-parasite interactions. To reach a wide range of researchers working on the epidemiology, transmission, and other aspects of malaria, the models have been critically analysed, so that it will be useful in understanding and classifying the numerous between-host models in this area. This may help mathematicians to further develop suitable models, and biologists and public health professionals to adopt better strategies for controlling the disease.

### Model basics

In epidemiological compartment models of infectious diseases, transmission of infectious agents in the host population is the fundamental process to be described. When a pathogen appears in a host community, it partitions individuals in the community into categories depending on parasite density inside them and the type of infection. These categories or compartments are represented by standard notation of *S-E-I-R *after the pioneering work of Kermack and McKendrik [16]. In a simple form they are as follows: the first group consists of the fraction of host population that is Susceptible (*S*) to infection; then comes the Exposed (*E*) class - the fraction of population whose individuals are infected by the pathogen, but not capable of passing on the infection to others during a *latent period *(Table 1). The next is *I *class or Infectious individuals, who give rise to more infected individuals through interaction with the Susceptibles. Finally, those individuals who recover from the infection make up the *R *class.

There may be variations in the compartment structure depending on the type of disease. For example, the *I *class of individuals may not recover at all and die; *R *can consist of individuals, who recover with temporary or permanent immunity, thereby further subdividing the epidemiological compartments. Using these notations, eight classes of compartmental models are possible - *SI, SIS, SEI, SEIS, SIR, SIRS, SEIR *and *SEIRS *[50]. For example, in an *SEIRS *model, a fraction of the susceptible (*S*) population gets exposed (*E*) to infection, a part of which then becomes infectious (*I*). Some from the *I *class recover from the disease, and become part of the *R *class with temporary immunity. When immunity is lost, they become susceptible to pathogen attack again, and enter the *S *class. The *Plasmodium *parasite requires both human and mosquito for its life cycle to complete, and the infection is transferred between susceptible human individuals through the bite of infected mosquitoes, which acquire infection through a blood meal from infected humans. In malaria models, therefore, these compartments have been applied to both human (host) and vector (mosquito).

**Figure 1:**
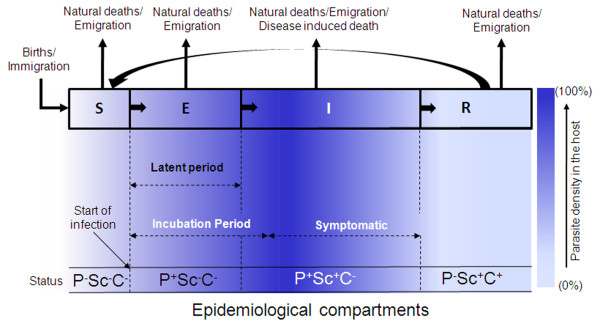
**Epidemiological Compartments separating different stages of infection and parasite density in a population**. S, E, I and R represent Susceptible, Exposed, Infected and Recovered fraction of the population respectively. Arrows on the top indicate different ways of population loss and transfer of population from one compartment to another. Different periods (Latent, Incubation, Symptomatic) characteristic of infection are shown by dotted arrows. The bottom panel shows the status of clinical markers for each compartment - PCR (P), Sero-conversion (Sc) and Cellular immunity (C) (positive or negative). Colour Bar indicates the density of parasites in host in different compartments (0-100%). See text and Table 2 for details.

Epidemiological compartments, for an *SEIRS *model, separating different stages of infection and parasite density in the host population, are shown in Figure 1. Different stages of infection, which are significant to the dynamics of transmission, are shown in the transfer diagram at the top of Figure 1. The level of infectious agent that replicates inside a host may develop from small inoculums to a higher level, and later decline and/or disappear altogether as it passes through these compartments (shown by blue colour scale in Figure 1). *Latent period, Incubation period *and *Symptomatic period *(Table 1 for definitions) are also shown in Figure 1. In many cases of infection, the *incubation period *and *latent period *are not the same [12]. Appearance of symptoms is important for case diagnosis and treatment. But *Asymptomatic *infections are commonly observed in humans and require clinical validation. The status of the clinical markers (presence/absence indicated by +/-) for diagnosis of each compartment is denoted by P (PCR), Sc (Sero-conversion) and C (Cellular immunity) [51-53], and are shown in the bottom panel of Figure 1 (Table 2 for details). Starting from the basic Ross model many transmission models of malaria have been developed by considering regulation of the passage of the human host and mosquito vector through these epidemiological compartments as a function of the host and parasite-specific factors, their interactions, and external environmental variables.

**Table 2:** Clinical markers for diagnosis

**Polymerase Chain Reaction (P):**	For identification of malaria parasites in blood, Polymerase Chain Reaction (PCR) is now a common and often vital technique, which amplifies a minute amount of DNA of the parasite across several orders of magnitude, generating thousands to millions of copies of a particular DNA sequence [51,52]. Due to absence of parasite in susceptible (*S*) and recovered (*R*) classes PCR indicates negativity whereas for exposed (*E*) and infected classes (*I*) PCR shows positivity.
**Sero-conversion (Sc):**	To determine antibody positivity as a result of infection or immunization, the clinical technique Serology is used. The development of detectable specific antibodies to microorganisms in the blood serum (Sero-conversion) is a reliable indicator for different infectious diseases including malaria [53]. Before Sero-conversion, the blood test shows Sero-negativity for the antibody (in susceptible and exposed classes); after Sero-conversion it shows Sero-positivity for the antibody (in infected and recovered classes).
**Cellular immunity (C):**	Parasite infection lead to development of specific memory immune cells (T-cells and/or B-cells), which is detected positive through clinical diagnostic of cellular immunity (C) in the recovered class.

### Hierarchy of malaria models

**Figure 2:**
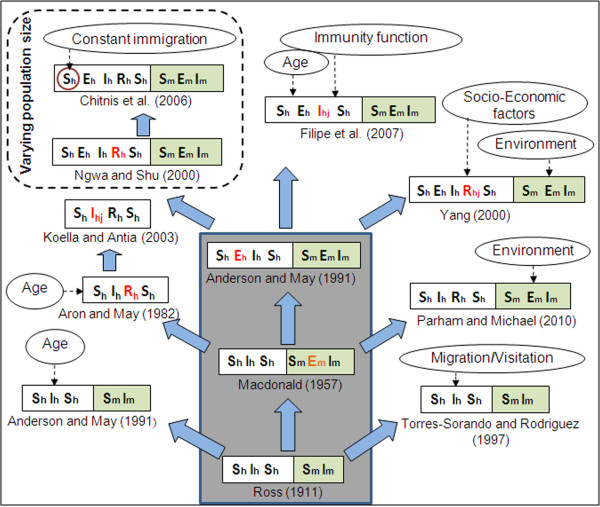
**Evolution and grouping of different types of SEIR malaria models**. Subscripts 'h' and 'm' stands for human and mosquito. Double-folded boxes are for both human & mosquito population, and single fold boxes are only for human. First time addition of a new compartment is shown in red. The subscript 'j' (= 1, 2, 3) indicates further subdivision of the corresponding compartment. Three models inside the big grey box are considered as the Basic malaria models in this paper. Dotted arrows show the incorporation of complex factors in different models or specific compartment (red circle). Total population size is constant for all models, except the ones inside the dashed box.

Even for the restricted set of deterministic Ordinary Differential Equation (ODE) models of epidemiological compartments being considered in this article, summarising hundred years of extensive theoretical work on malaria modelling with the incorporation of ever-increasing complexities, have the possibility of unintentional bias, omissions, and under-representations. Keeping this in mind, an attempt has been made here to elaborate the evolution of these models by considering some representative mathematical models that include the increasing complexities of host-vector-parasite interactions. The word "hierarchy of models" used here (shown here as a tree in Figure 2), is based on the undisputed fact that the start of the tree is the model by Ronald Ross [36-39], and its highly significant improvement, with focus on application in mosquito eradication, by George Macdonald [40]. The rest of the models have been grouped here purely on the basis of increasing complexity of the epidemiological compartments in the host and vector populations, and hence the temporal order is not conserved. The three basic models, based on which other models were developed, are shown as the grey trunk of the tree.

The epidemiological compartments are kept in double-fold boxes in Figure 2. The human classes (*S_h_, E_h_, I_h_, R_h_*) are in the left fold and the mosquito classes (*S_m_, E_m_, I_m_*) in the right. In general, the human classes in malaria infection end with the susceptible (*S_h_*) class, but the mosquito populations die of infection and hence it can only go up to the infected class (*I_m_*). In models where there is only a single-fold box (for the human classes), the effect of vector is introduced through their *vectorial capacity *of infection (Table 1 for definition). The newly introduced compartment over the earlier one is shown in red. The effects of different complex factors, such as age, immunity, environment and socio-economic, in different models or specific compartments (red) are shown by dotted arrows.

Ronald Ross in his first mathematical model of malaria used the word "pathometry" to mean "quantitative study of a disease either in the individual or in the community" [36]. Ross, through his model, showed that reduction of mosquito numbers "below a certain figure" (*Transmission threshold*) was sufficient to counter malaria - a concept far ahead of his time. After about 40 years, George Macdonald [40], in the 1950s, reasserted the usefulness of mathematical epidemiology based on 20 years of fieldwork. He modified Ross's model by integrating biological information of latency in the mosquito due to malaria parasite development, and implicated the survivorship of adult female mosquito as the weakest element in the malaria cycle. This provided a rationale for a massive World Health Organization (WHO)-coordinated campaign, which focused on using the insecticide dichlorodiphenyltrichloroethane (DDT) that killed mosquitoes, for the elimination of malaria transmission among 500 million people in Africa [54,55]. Latency of infection in humans was introduced by Anderson and May [12] in Macdonald's model making the additional "Exposed" class in humans.

Researchers have modified the basic Ross model to explain the effect of age structure of prevalence [12], migration and visitation of people [48]. Several models were also put forward after Macdonald's model by combining additional complexities of human immunity, parasite diversity, and resistance, to explain large amounts of epidemiological data collected in Africa and other parts of the world [17,56,57]. They were fairly successful in describing region-specific incidence data. Thus, all other models shown in Figure 2, and discussed in this paper, are developed from these three basic models by incorporating different factors to make them biologically more realistic in explaining disease prevalence and prediction. The only new class that is added in humans is the recovered (*R_h_*) class, which incorporates a time dependent immunity developed on recovery from infection, before being transferred to the susceptible (*S_h_*) class again. The major advantage of these early models was to provide a suitable control strategy through the *Transmission threshold *criterion, which is based on the reproductive capacity of the parasite, and termed as *basic reproductive number, R_0 _*(Table 3 for details). Even though the concept of threshold was first introduced by Ross, it originated from Fisher's "*net reproductive value*" for a parasite [58]. From its inception the concept of *R_0 _*is widely discussed in any study on population biology of a parasite [59-61]. The basic results of all these models can also be described by estimating the basic reproductive number (*R_0_*).

**Table 3:** Definition of basic reproductive number (R_0_)

**Basic Reproductive Number (*R_0_*)**	The basic reproductive number, *R_0_*, is the average number of successful offspring that a parasite is capable of producing [12,61]. For the compartmental models of malaria this is defined as the number of secondary cases of malaria arising from a single case in an otherwise uninfected population [40], and can be thought of as a measure of the intensity of transmission. The estimation of basic reproductive number (*R_0_*), from both models and data has been discussed by several researchers over the years [12,59-61,125]. Malaria can spread in a population only if *R_0 _*> 1 (epidemic), but when it is maintained in a population without the need for external inputs *R_0 _*= 1 (endemic). A disease free population is possible when *R_0 _*< 1. These threshold conditions of *R_0 _*may not hold for stochastic models. In that case the disease may go extinct even for *R_0 _*> 1, depending on the magnitude of stochastic fluctuations around the endemic equilibrium state [12].

In the following sections, starting from the details of the basic models, the representative mathematical models in each group along with their underlying features are discussed, and their specific contributions are reviewed.

## Important features and comparative analysis of mathematical models

### Basic models

The three basic models, shown in the grey trunk of the tree in Figure 2, are given in Table 4. The first column of the Table gives the mathematical model; the basic reproductive number, *R_0 _*- is given in the second column; and, the parameters (with range of values used in literature) are described in the last column. These basic models used the simplest scenario by incorporating only two critical features for predicting malaria progression in the host and vector populations - epidemiological compartments in the populations, and latency periods of pathogen in the mosquito and human. The population size of human is kept constant (unity) in all three basic models.

**Table 4:** Basic malaria models - (a) Ross Model, (b) Macdonald Model, and (c) Anderson-May Model, with corresponding basic reproductive number (R_0_) and parameter descriptions

Models	*R_0_*	Parameters and their values [12,36,40,44,56]
(a) **Ross model **[36]		*a *: Man biting rate [0.01-0.5 day^-1^] *b *: Proportion of bites that produce infection in human [0.2-0.5]
(b) **Macdonald model **[40]		*c *: Proportion of bites by which one susceptible mosquito becomes infected [0.5] *m *: Ratio of number of female mosquitoes to that of humans [0.5-40] *r *: Average recovery rate of human [0.005-0.05 day^-1^]
(c) **Anderson and May model **[12]		*μ_1_*: Per capita rate of human mortality [0.017 year^-1^] *μ_2_*: Per capita rate of mosquito mortality [0.05-0.5 day^-1^] *τ_m_*: Latent period of mosquito [5-15 days] *τ_h_*: Latent period of human [10-100 day]

Ross introduced the first deterministic differential equation model of malaria by dividing the human population into *susceptible *(*S_h_*) and *infected *(*I_h_*) compartments, with the *infected *class returning to *susceptible *class again leading to the *SIS *structure. The mosquito population also has only two compartments (*S_m_, I_m_*), but they do not recover from infection due to their short life span, and thereby follow the *SI *structure. Time evolution of the fraction of individuals in the infected classes (*I_h_, I_m_*) is studied using two differential equations - one each for the human and mosquito (Table 4). It is clear that the parameters, *m, a, b*, and *c*, that contribute to the increase of *R_0 _*in this model, are related to mosquitoes and humans, and any change in them can significantly affect malaria transmission. Increasing mosquito mortality and reducing mosquito biting rate can reduce *R_0_*. The Ross model outlines the basic features of malaria transmission, and puts the main burden of transmission on mosquito-specific features, thereby paving the way for mosquito-based malaria control programmes.

The malaria parasite spends approximately 10 days inside a mosquito during its life cycle. The simple Ross model did not consider this latency period of the parasite in mosquitoes and their survival during that period. This resulted in the model predicting a rapid progress of the epidemic in human, and a higher equilibrium prevalence of infectious mosquitoes. Macdonald considered this latency period (*t_m_*), and introduced the *Exposed *(*E_m_*) class in the mosquitoes [40]. Therefore, in this model (Table 4), the mosquito population is divided into three compartments (*SEI*), and the model studies the time evolution of the exposed (*E_m_*) and infected (*I_m_*) classes in mosquito. The *R_0 _*for this model is consequently scaled down with increasing latency period.

**Figure 3:**
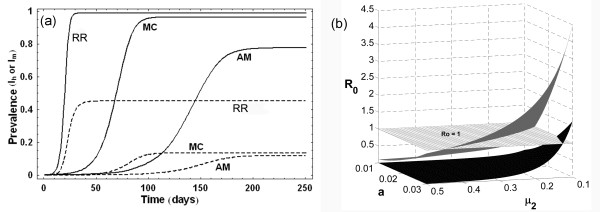
**(a) Prevalence curves of human (I_h_: solid line) and mosquito (I_m_: dashed line) populations in Ross (RR), Macdonald (MC) and Anderson-May (AM) models**. Parameters used are: *a = 0.2 day^-1 ^, b = 0.5, c = 0.5, m = 20, r = 0.01 day^-1^, μ_1 _= 0.017 year^-1^, μ_2 _= 0.12 day^-1^, τ_m _= 10 days, τ_h _= 21 days*. (b) Variation of basic reproductive number (*R_0_*) with mosquito biting rate (*a*) and mosquito mortality rate (*μ_2_*) in Ross model (grey surface) and Anderson-May model (black surface). The surface of *R_0 _= 1 *is shown as gridded white plane.

In a natural extension to the Ross and Macdonald's models, Anderson and May considered the ~21 days latency period of the parasite in humans, and introduced the *Exposed *(*E_h_*) class in human population in their model [12]. This divided the host population into three compartments (*S_h_, E_h_, I_h_*), along with that in the mosquito population (*S_m_, E_m_, I_m_*). This, therefore, is a *SEIS *model for the human population, and the model consists of four differential equations (Table 4) describing the time evolution of both the exposed and infected classes for humans and mosquitoes (*E_h_, I_h_, E_m_, I_m_*). The *R_0 _*for this model is further reduced due to inclusion of human latency period. A comparative study of Ross (RR), Macdonald (MC), and Anderson-May (AM) models for the prevalence of infected humans and mosquitoes (*I_h_, I_m_*) is shown in Figure 3a. The figure shows that inclusion of the latency periods of parasites in humans and mosquitoes not only reduces the long term prevalence of both *I_h _*and *I_m _*(RR being the highest and AM the lowest); the rates of progression to these final infected populations are also reduced. Even with this minimal complexity, these basic models can give some idea of the effect of different types of interventions on disease transmission dynamics, which is discussed in the next section.

### Predicting the effects of interventions in the basic models

The parameters mosquito density (*m*), biting rate (*a*) and mosquito mortality rate (*μ_2_*) are important in regulating the fraction of human population that will enter into the exposed (*E*) and infected (*I*) classes. The most important fact for any epidemiologist or public health person is to have an idea about the relative effects of interventions in these parameters to the intensity of transmission, the measure of which is *R_0_*. Given the expressions of *R_0 _*in all three models in Table 4 it is clear that the square dependence of the biting rate '*a*' implies that halving the biting rate is more effective than halving the coefficients '*b*' or '*c*' in all three models Thus, reducing the biting rate (by using bed nets, or any other method) will be an effective method of controlling the transmission. But this is not so obvious for all parameters. For example, the relative effect of reducing the adult mosquito mortality (*μ_2_*) in comparison to biting rate (*a*) is different in these models due to the presence of the exponential function of *μ_2_*. Figure 3b shows this with the Ross and Anderson-May models, where the variation of *R_0 _*is plotted with changes in two parameters, biting rate (*a*) and adult mosquito mortality (*μ_2_*) with other parameters as per Table 4. Due to higher disease prevalence predicted by Ross model (see Figure 3a), the *R_0 _*surface is also higher compared to that of the Anderson-May model. The surface of *R_0 _*= 1 shows that onset of epidemic happens at higher values of parameters in Ross model compared to Anderson-May model. These results indicate that compared to the reduction in biting rate '*a*', reducing the length of life of adult mosquitoes is most effective in decreasing malaria cases, in the latter two of the three basic models. As mentioned before, these model results provided rationale for control of malaria transmission through the mosquitoes, using insecticides (DDT) and insecticide-impregnated bed nets, since they affect *m, a*, and *μ_2_*. Thus, even at this low level of complexity, these models had been successful in describing the factors that influence the transmission of the disease, which were useful in control and eradication of malaria from many countries of the world.

### Complex models

Over and above the simple scenario described in the basic models, many other factors such as, host factors, demographic heterogeneity, geographic distribution of populations, rules of social interactions, climate and environmental influences, and the ecology of the area play important roles in the development of malaria in space and time. Age-specific host immunity, parasite diversity, DDT and drug resistance dynamics, vector population dynamics, effect of global warming are also interacting factors and variables that influence disease dynamics at different scales. There has not been *A MODEL *that has been able to incorporate all factors and variables because of the overwhelming complexity of the system. Also, a model's utility may not always lie in its mathematical analysis or incorporating finer details. The ability to base it on relevant details and ask specific questions that can be tested, are the hallmark of useful models. Along with fitting the past data and predicting the future, it should also be able to point to areas where data needs to be generated in order to increase our conceptual grasp. Such improvements in modelling generally occur in multiple steps, one leading to the other, as more information become available. The next section elaborates on some representative next-generation mathematical models that evolved from the above-mentioned basic models, and includes the increasing complexities of host-vector-parasite interactions. Specifically, the factors considered here are - (i) Age and immunity, (ii) Host-Pathogen variability and resistant Strains, (iii) Environmental factors, (iv) Social and economic factors, and (v) Migration and visitation.

#### Age and immunity

Malaria burden differs depending on age and gender in humans. In African children, most malaria deaths occur under the age of 5-years. As a result of continuous exposure and the ability to develop a degree of immunity to the disease, older Africans have reduced risk. Outside Africa, where continuous exposure does not occur, the disease burden extends into adulthood [1]. Age and immunity, therefore, are known to be important inter-related factors for transmission of malaria in a population. The importance of incorporation of immunity in malaria models is aptly described by Koella [56] - "*Incorporating immunity into malaria models is important for two reasons. First, the neglect of immunity leads to unrealistic predictions. Incorporating immunity can help to make models more realistic. Secondly, modelling immunity, and in particular the effect of vaccines, can help to predict the outcome of vaccination programmes"*. A number of epidemiological studies [41,43,44,57,62] have focused on this important aspect by including immunity and age structure of the human community in the models. In this scenario, the infection moves differentially within different age groups based on their immune status, and also with time.

Age structure was included by **Anderson and May **[12] in the simple Ross model by considering the human population density in the *I_h _*class as a function of age (*α*) and time (*t*) as,(1)

where, *N(α) *denotes the population density of human at age *α*, and  is the mosquito density. Other variables and parameters are as described in Table 4. In this scenario, the infection moves differentially within different age groups and also with time. With the inclusion of a simple force of infection (Table 1), which represents the per capita rate of acquisition of infection based on *N*, , '*a*' and '*b*', this simple model can improve upon the basic models to include the age dependence of infection in human community. But the dependence, as predicted by this model, did not match well with observed trend in prevalence with age [63], and it was clear that the interaction of age and immunity needs to be modelled more explicitly.

Immunity can be included in a model in two ways - by considering a separate Immune class (*R_h_*) in humans, and by incorporating an Immunity function in existing models. Some models (Dietz *et al *[57], Aron [43], Ngwa and Shu [64], Ngwa [65], Chitnis *et al *[32,66], Yang [67,68]) have introduced a separate *immune class *in their models, whereas, some others (Fillipe *et al *[44] and [69-72]) have used complex *immunity functions *in their model. Assuming that the malaria immunity is not permanent, Dietz *et al *[57] first proposed a model considering seven compartments of human. The effect of mosquito was introduced through *vectorial capacity*. In this model a person may either recover from the infected class (*I_h_*) and directly return to the susceptible class (*S_h_*), or become re-infected through a temporary immune class (*R_h_*). The model has shown a good fit to the data obtained from northern Nigeria. The changes on each compartment in this model were presented using difference equations. The differential equation based models that incorporate immune classes are discussed below.

##### Immune class

Generally immunity is modelled by considering the fact that individuals are born susceptible to become infected at a rate of *h *infections per year, but they subsequently recover and acquire immunity at a slow rate. If immunity is temporary and lasts only for *τ *years (in absence of new infections), then they again become susceptible to infection. Immunity is also boosted by new infections. In this simple scenario [73], the average per capita rate of loss of immunity *γ*(*h, τ*) is given by

**Figure 4:**
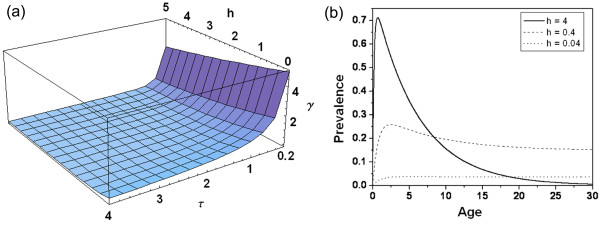
**(a) Variation in loss of immunity (*γ*) with force of infection (*h*), and immunity period (*τ*) in absence of new infections**. (b) Age-Prevalence curve simulated from Aron-May model for three different levels of force of infection (*h*). Other parameter values are *r = 0.8, q = 0.2*, and *τ = 5*.

The range for *h *is estimated between 0 to the order of 10^3 ^[74,75], and the upper limit of τ has not been reported so far [56]. The variation of *γ*(*h, τ*) with force of infection (*h*) and period of immunity (*τ*), in absence of new infections, is shown in Figure 4a for a small parameter range to highlight the relative effects of changes in *h *and *τ*, as the major variation of *γ *is observed only for low values of *h*. Due to continuous exposure, in high endemic zones where force of infection (*h*) is very high, the rate of loss of immunity is nearly zero. Figure 4a clearly shows that the rate of loss of immunity (γ) is faster at low *τ *values, whereas for the force of infection (*h*), it decreases slowly. For a fixed *τ, γ *decreases monotonically with force of infection (*h*), implying reduced rate of transformation from recovered class to susceptible class due to increase in force of infection. It is further reduced if the period of immunity (*τ*) is increased. Some of the models that have considered the immune class are discussed below.

In a population that has reached its equilibrium pattern of infection, time can be represented through the age (α) of the cohort. Considering this, Aron and May [41] proposed an age-specific immunity model with a new compartment - Immune (*R_h_*) - in humans. This model, thus, consists of three compartments in humans: Susceptible (*S_h_*), Infected (*I_h_*) and Immune (*R_h_*), and is a SIRS model. This model is shown in a single-fold box for host in Figure 2, because the effect of mosquito is introduced only through the force of infection, *h*. The infected individuals can recover at a rate *r *to become susceptible again, or may acquire immunity at a slow rate of *q*. This simple model of immunity, incorporates the immunity factor by adding an extra term *γ*(*h, τ*) *R_h _*- of people who lose immunity - in the susceptible class, and subtracting the same from the immune class in equation (2).(2)

Solution of equation 2 shows how the prevalence of infection varies with the age of human. Figure 4b shows the prevalence of infection (*I_h_*) with age at three different forces of infection (*h*). At higher infection (*h = 4*), *I_h _*rises rapidly with age in young infants and children, attains a peak, and then declines in the older children to reach a low level in adults. Prevalence in adults decreases due to the increase in immunity. For low *h *(*h = 0.04*), this dependence on age is negligible. This model predicts that the prevalence rises quickly in early childhood and declines slowly into adulthood in highly endemic areas, due to slow acquisition of immunity with age/time. Interestingly, the prevalence among adults is highest when *h *is in an intermediate value. The adult crossover of the age-prevalence curve with increasing *h *resembles the pattern of acute infection described by Boyd in tropical Africa [63].

Inclusion of the "Recovered" class with immunity of the host has been the source of many later models that considered other variations in host-pathogen interactions [12,56]. A few models are mentioned below, which along with introducing the "Recovered" class in humans in the Anderson-May (AM) model, also differ in some of the critical assumptions from the models discussed so far. One of the features that has been consistently followed in all the models discussed above is the constancy of population size. Mortality and migrations are major factors in changing the population size in an area and the inclusion of varying population size in the model makes them more realistic.

Ngwa and Shu proposed an immunity model in which disease related death rate is considered to be significantly high, and the total population is not constant (shown inside the dashed box in Figure 2). The Ngwa-Shu model [64] model consists of four compartments in humans - Susceptible (*S_h_*), Exposed (*E_h_*), Infected (*I_h_*) and Immune (*R_h_*) - and three compartments in mosquitoes - Susceptible (*S_m_*), Exposed (*E_m_*), and Infected (*I_m_*) (see Additional file 1 Table S1). Mathematical analysis of the model shows that the Basic Reproductive Number, *R_0_*, can describe the malaria transmission dynamics of the disease, where a globally stable disease-free state exists if *R_0 _*< 1, while for *R_0 _*> 1, the endemic equilibrium becomes globally stable. This model explicitly shows the role of inclusion of demographic effects (net population growth) in predicting the number of fatalities that may arise as a result of the disease. In a similar theme, Chitins *et al *[32,66] included constant immigration of susceptible human population, (see Additional file 1 Table S2). Considering immigration of people and excluding direct human recovery from the infectious to susceptible class (as is considered in other models here), they showed that the population approaches the locally asymptotically stable endemic equilibrium point, or stable disease-free equilibrium point, depending on the initial size of the susceptible class.

Immunity can be described as a continuum of different levels of protection rather than a single class, as anti-malarial immunity develops slowly among people exposed to continuous and intense malaria transmission. Yang [67] divided the immune class (*R_h_*) in human population into immune (*R_h1_*), partially immune (*R_h2_*) and non-immune but with immunologic memory (*R_h3_*), with each class having differential immunity (see Additional file 1 Table S3). The mathematical analysis of Yang model shows that the effects of these three types of immune responses lead to delay in the reappearance of the individuals, who already had experienced malaria, to the susceptible population. Hence the community under high threat of malaria (high *R_0_*) shows low prevalence of individuals with asexual blood-stage infection and without infectious gametocytes, whereas, the same community is relatively free of severe infection due to the increase in immunity by re-infection.

##### Immunity functions

Due to lack of confirmed markers of immunological protection, different processes that determine the immunity acquisition to clinical disease and to asymptomatic carriage of malaria parasites are poorly understood. The models discussed in the earlier section consider the immune individuals as a separate class, with no consideration of the types of processes that drive acquisition of immunity and its role in disease progression. In an insightful approach, Filipe *et al *[44] introduced three age-specific "immunity-functions" in their SEI model for the human host, in which the infected humans are divided into three classes - infected with severe disease (*I_h1_*), asymptomatic patent infection (*I_h2_*), and infected with undetectable parasite density (*I_h3_*). The effect of mosquito density is incorporated through the force of infection (*h*). The dynamics of transmission of infection in this model is given in Additional file 1 (Table S4).

The three immunity functions (IF) introduced in the Filipe model are - (i) Reducing the susceptibility to clinical disease, *ϕ *(IF1), (ii) speeding up of the clearance of detectable parasites, *r_A _*(IF2), and (iii) increasing tolerance to sub-patent infections, *r_ij _*(IF3). These functions depend on age and disease transmission intensity (i.e., *Entomological Inoculation Rate*, see Table 1) in a complex manner. They base their model assumptions on the fact that the rates at which both types of immunity - clinical and anti-parasite - develop are different. Details of the immunity functions are given in Additional file 2. All these processes have widely varied time scales, which make the disease transmission in this age-structured population quite complex. The first two types of immune functions reproduced the epidemiological age-prevalence curves seen in empirical data better. The third one i.e. the tolerance to sub-patent infections, is not required to explain the empirical data.

**Figure 5:**
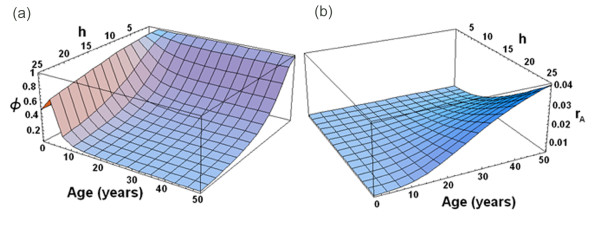
**Response of immunity functions: (a) susceptibility (*ϕ*) to develop clinical disease; and (b) rate of clearance of detectable parasites (*r_A_*), to variation in force of infection (*h*) and age**.

Susceptibility to clinical disease (effect of IF1) develops early in life and then decreases with age, and is inversely proportional to the inoculation rate (Figure 5a). Anti-parasite immunity, on the other hand, develops later in life and results in more rapid clearance of parasite. Figure 5b shows that the rate of recovery from detectable parasite (effect of IF2) increases with age and force of infection (*h*). These figures clearly show that these two distinct acquired immunity processes are required to demonstrate: (i) reduction in clinical susceptibility and (ii) a parasite immunity process that develops substantially in later life, and, which increases the rate of natural recovery. In addition, this also explains the duration of clinical and parasite immunity from the age-prevalence pattern. To show the efficacy of different intervention strategies more complex models have been developed in this area [69-72]

#### Host-pathogen variability and resistant strain models

The basic models assume homogeneity in the host and parasite populations in terms of their response to the process of transmission of infection. They consider all the individual hosts and parasites in the population to have an equal chance of developing disease or becoming immune or transmitting infection. However modern application of molecular typing methods has shown that there exist diversity among host and parasites in responding to infection. Further, long term and indiscriminate use of insecticides (DDT) and drugs (quinine and chloroquine) brought forth the hitherto neglected issue of heterogeneity in vector and parasite phenotypes and genotypes. The evolutionary consequences of these interventions had serious negative impact in malaria control. Most models of population heterogeneity and resistance consider *within-host *processes. Many mathematical models have been developed with pathogen population structure and heterogeneous host population to describe variable antigenic response, immune selection, pathogen strain structure [76-78]. Inclusion of evolution of drug resistance, along with other factors, in the models can help in the design of rational strategies for the control of drug resistance [79-86].

Several resistant-strain models have been developed based on evolution of drug resistance through host immunity [82,85], and by considering the practical implications of the artemisinin combination therapy (ACT) drug policies adopted by many countries [84]. Population genetic considerations of the *cost of resistance *(Table 1), are also included in this type of models [81,87]. More recent work elaborates the complexity of the process of drug resistance by considering the interaction of several environmental, pharmacological and genetic factors [86]. These models are important as they address phenomena critical to public health, i.e., the evolution of drug resistance in malaria parasites.

In general, these resistant-Strain models divide the infected host population (*I_h_*) into two compartments, i.e., infected by drug-sensitive strain and drug-resistant strain of the parasite.

The model, proposed by Koella and Antia [82], further divides the host population infected by drug-sensitive strain into two compartments - *treated *and *untreated*. So this model consists of five compartments of human: susceptible (*S_h_*), sensitive, infected, and treated (*I_h1_*), sensitive, infected, and untreated (*I_h2_*), infected with the resistant strain (*I_h3_*), and the recovered (*R_h_*). The role of mosquito vector is included through inoculation rates of sensitive and resistant parasites. The formulation of the model is described in Additional file 1 (Table S5). The primary prediction of this model indicates that there is a threshold proportion of people (*f_c_*) among the infected and treated (*I_h1_*) classes, below which resistance cannot spread, and above which resistance will eventually become fixed in the population. The threshold level *f_c _*is defined as:

where, ∈ is termed as the "Effectiveness of treatment", i.e., the ratio of the duration of infection for the untreated and treated sensitive parasites, and Γ is the *cost of resistance*. Thus, these two parameters (Γ and ∈) regulate whether drug sensitive or resistant parasite will be dominant in the population. The model also shows that, in the absence of drug or treatment, the fitness of resistant parasite reduces with respect to sensitive parasite; otherwise both the parasites have identical properties. In this case, sensitive and resistant parasites cannot co-exist.

#### Environmental factors

The epidemiology of the host, vector, and pathogen for malaria necessitates consideration of the conditions that increase the mosquito population density. Modelling the dynamics of mosquito populations to increase understanding of malaria transmission across a range of environmental conditions, including climate change, is an important and emerging research area. The basic reproductive numbers (*R_0_*) for the basic models depend crucially on the parameters related to mosquito density. Environmental factors, such as temperature, humidity, rainfall and wind patterns have great impact on mosquito reproduction, development and longevity and the parasite survival in its life cycle in mosquito. It is known that mosquito breeding is influenced by temperature - a change in temperature from 12°C to 31°C reduces the number of days required for breeding from 65 days to 7.3 days [88]. The sporogony of the parasites in vector is completed in 55 days at 16°C, which reduces to 7 days at 28°C [89]. With recent surge of interest on the effects of global warming on malaria incidence, modelling the effects of environmental factors in malaria transmission has become quite relevant and topical [6-8,90,91]. As humidity is conducive to mosquito growth, rainfall and stagnant water bodies also influence mosquito density.

The environmental impact on the transmission of malaria is therefore studied primarily by modifying the mosquito population dynamics. Influence of temperature and humidity change on the rate of transformation from juveniles to adults in the susceptible class of adult mosquitoes has been modelled [88]. In addition, several mathematical studies have been performed to simulate the effect of environmental variability in the abundance of mosquito populations such as, random fluctuation in the form of colour noise in infected mosquito dynamics of Ross model [92], periodic or noisy form of the force of infection [12,41,92]. Several studies have also included the effect of environmental fluctuations in diverse ways [2,5,67,68,93,94] with the aim to develop realistic and validated malaria modelling frameworks that are able to identify the crucial linkages between pathogen transmission processes and climactic factors.

In a recent study, Parham and Michael proposed a model [5], to study the dynamics of the mosquito population by considering simultaneous effects of rainfall and temperature. The model consists of three compartments in humans (*S_h_, I_h_, R_h_*) with fixed duration of latency, and three compartments in mosquitoes (*S_m_, E_m_, I_m_*) (see Additional file 1 Table S6). Different environmental factors are introduced in this model through parameters related to mosquitoes. The birth rate of adult mosquito is considered to be a function of rainfall and temperature, whereas, mosquito mortality rate, biting rate, duration of sporogonic cycle and survival probability of infected mosquitoes over the incubation period of the parasite are considered to be dependent on temperature variation. The major finding of this model is that changes in rainfall patterns not only influence vector abundance, but also strongly govern malaria endemicity, invasion and extinction. However, when sufficient rainfall exists to sustain vector development and survival, then the temperature affects the pathogen life cycle, and has stronger influence on the rate of disease spread.

#### Social and economic factors

Malaria risk is highly dependent on the socioeconomic conditions of the host population. It is now fairly evident that "*as a general rule of thumb, where malaria prospers most, human societies have prospered least*" [95]. Poverty is largely concentrated in the tropical and subtropical zones, and that is where most malaria transmission is observed. The extent of the correlation suggests that malaria and poverty are intimately related. In most endemic areas of malaria, changes in social and economic conditions are considered to be far more important than temperature shift [68]. The economic and social burdens from factors such as fertility, population growth, premature mortality, misdiagnosis, inflicted by the disease on the society have been studied by many authors [95-100]. Given the nature of the factors, most investigations are case studies, and there are only few differential equation based models that incorporate socio-economic structure.

Using a mathematical model (see Additional file 1 Table S3), Yang showed how the basic reproductive number (*R_0_*) of malaria transmission changes with global warming and local social and economic conditions [68]. In this model good, intermediate and poor economic conditions among human community have been considered and each condition is further divided into three temperature zones. A host of factors control disease transmission rates in this model such as, differential immunity, endemicity, resistance, economic conditions and temperature dependence of mosquito development. These lead to different *R_0 _*for three temperature zones with different socio-economic structures. These modelling results point out the requirement of proper management of the surrounding environment, along with good health care system, in disease transmission. From the point of view of designing field research, it is shown in a mosquito based model [101], that the effectiveness of malaria control through different types of intervention methods (insecticide-treated nets and indoor residual spraying) can have differential protection, with the former being more protective. The socio-economic scenario for large scale deployment of interventions at the population level has also been addressed using modelling studies [102].

#### Migration and visitation

One of the important reasons for the failure of strategies to eradicate infectious disease is because of their neglect of the mobility patterns of the host. The importance of the role of human migration is evident in the recent increase in malaria incidence not only in the endemic zones, but also in zones where malaria had been eradicated [17,103-105]. Mainly two types of mobility patterns that can spread the infection to newer areas, are considered - *migration*, i.e., when the people move from one region to another with no returns; and *visitation*, when the people return to their original region after visiting other regions.

The effects of migration and visitation on transmission of malaria were shown by Torres-Sorando and Rodriguez by modifying the basic Ross model to include space that is fragmented into *A *number of patches [48]. Only humans are assumed to move among the patches and mosquitoes are evenly distributed. The equations describing the system are shown in Additional file 1 (Table S7i). The number of infected humans and mosquitoes in patch *i *(*i = 1, 2,....., A*) at time *t *are, *I_hi_*(*t*) and *I_mi_*(*t*), respectively, and *e_ij _*is the fraction of humans that migrate per unit time from patch *i *to patch *j*, and do not return. So, to incorporate the effect of migration in this model, an extra term related to infected people due to movement between the patches, i.e., is added to the human dynamics equation. For visitation, the individuals visit from patch *i *to patch *j *during a time, which is a fraction *T_ij _*of the time unit, and then return to their patch of origin. The number of newly infected people added during visitation is,. The corresponding equations for the visitation model are shown in Additional file 1 (Table S7ii). The model results show that increase in mobility between patches enhances the persistence of the disease. Even though migration of humans does not change the equilibrium prevalence, equilibrium is reached faster for higher levels of migration. When there is visitation, the equilibrium prevalence increases with visitation time, and the time to reach the equilibrium decreases with increase in the intensity of visitation.

The elaboration of different approaches to model malaria transmission in populations, as described in Figure 2, is given in the earlier sections. As mentioned earlier, only few models have been discussed above as examples to demonstrate the manner in which different factors and variables relevant to the host-vector-parasite biology have been incorporated in the basic models. Because of the overwhelming complexity of the disease system and its nonlinear interdependence on the environmental and socio-economic factors, there has not been one consensus general model where all factors are included. Linking the within-host and between-host dynamics of malaria has been one approach towards a comprehensive model [26,27]. Some effort has also been directed to develop software that include many factors for simulating the disease output [23,106].

### Stochastic models

Plasmodium life-cycle and mosquito population density are highly dependent on different internal processes and external environmental factors, which are probabilistic in nature. In many of the models referred above, stochasticity has been included in different ways. Even when the main structure of the compartments is similar to the differential equation based models, stochasticity has been included through individual variability in individual based models [24,27], and probabilistic variation in different variables and parameters of transmission processes and environmental factors [27,107-110]. Models integrating stochasticity with other factors such as, spatial contact structure and temporal forcing, also explain many interesting features of disease transmission [111,112]. Though not discussed in detail here, these models are powerful tools to explain complex interactions characterized by realistic descriptions.

## Data based statistical modelling

One of the major uses of a model is to fit past data and predict the future trend. This capability of a model improves the credibility of the underlying hypothesis of the model. In general epidemiological data represent the disease prevalence in an area over a period of time (time series data), and is given by the number of cases and number of infected persons. As has been enumerated in the earlier sections, this number, representing the prevalence of the disease, is the result of a large number of interacting nonlinear processes in host-pathogen interaction - both deterministic and probabilistic. These processes may have variable contribution to the development of the infection depending on the type of disease - a few may have larger contribution in the process, and others can be peripheral. Statistical modelling involves developing relationships between these factors in the form of mathematical equations. Statistical models that fit curves of past temporal prevalence of a disease, do not make any assumptions about the internal mechanisms that a mathematical model provides. This modelling approach involves application of a variety of elaborate statistical methodologies and tests, and has been extensively used in describing and predicting (*forecasting*) malaria incidence in different regions.

Several results from the models described in the previous sections have been validated with experimental data. Incorporating age and immunity, Aron [73] verified the results with the Garki project data [62]. To show the effect of socio economic conditions and environmental factors, Yang tested his model results with malaria data from three regions - (i) disease free but potentially under risk (Southeast Brazil: upper bound of temperature 20°C), (ii) disease at low endemic levels (Amazon region and Southeast Asia), and (iii) disease at higher endemic levels (Africa: lower bound of temperature 31°C) [68]. Filipe *et al *used the data from Northern Tanzania and north and south bank of river Gambia, to verify their model findings [44]. This kind of statistical modelling remains a highly preferred approach in quantitative malaria research to understand the disease incidence and the role of different factors [113].

One useful statistical approach to understand how disease prevalence changes based on other variables such as, temperature or rainfall, is the method of Regression modelling [114]. It involves techniques for modelling and analysis of the relationship between a dependent variable and one or more independent variables. Different variants of this method has been used to model malaria transmission in Africa through the Malaria Risk in Africa (MARA) project and in Europe [115], understanding the effect of childhood population data, climate averages and Normalized Difference Vegetation Index (NDVI) in Mali from 1960 [116], role of temperature and rainfall from meteorological stations and community-based parasitological survey in the prediction of malaria risk [117], effect of vector abundance, population immunity, on malaria incidence [118], predict the seasonal pattern of malaria in Kenya using NDVI [119] and to show evidence and causality between health and poverty in malaria prevalence [120]. Researchers have also used elaborate time series analysis models to show seasonality patterns in the malaria incidence [121-124].

With the availability of world-wide datasets on population distribution, global circulation, environmental factors, and parasitological prevalence, epidemiologists have now increasingly been interested in global modelling perspectives [89]. Such activities require, along with mathematical models, in depth statistical modelling techniques such as, bayesian inference using Markov chain Monte Carlo method, and multivariate statistical modelling techniques to generate maximum likelihood predictions for posterior probability of parasite distributions on the world map [6,125-127]. The most reassuring result from these global studies is that contrary to prevailing forecasts of global malaria expansion due to climate change, other natural and anthropogenic forces acting on the disease have actually resulted in a net reduction in transmission.

The above theoretical exercises depend crucially on the quality and availability of data and the methodologies used to build the models. They yield limited predictability and understanding due to factors such as, lack of information or excessive complexity. These models also evolve based on newer data and increased understanding of processes involved. Thus the data based analysis in the statistical modelling approach continues to be a major area in malaria research.

## Summary and outlook

A model is a mathematical abstraction of reality. The level of abstraction depends on the questions asked and the scale at which the underlying causative processes are studied. For example, in inter-host transmission of an infection, most molecular events in host-pathogen interactions (e.g., types of immune cells involved, parasite development inside the host, signaling pathways) are not considered. Many of these processes are condensed into a single parameter in the immune function or inoculation rate. In intra-host models, on the other hand, how the titers of the infective agent or related molecules change in an individual is studied, because that is what decides the diseased state of the host individual. In epidemiological models, intra-host processes between host-parasite-vector are neglected, but the host and vector population are subdivided in terms of the infection/diseased state (i.e., Susceptible, Exposed, Infected, and Immune/Recovered). These models aim to match their results with the available epidemiological data, where incidence of the infectious disease (and death due to it) determines the status of an epidemic in the population. Here, from the public health point of view, one is more interested in knowing if the infection will die out, or persist in a population through the important parameter *R_0_*. Yet, as more molecular studies are coming to the fore and both detection of infection and mode of infection propagation (genes, proteins, pathways, immune interactions) are elucidated, the epidemiological models also would need to consider these processes for inclusion as parameters or subclasses. This is clearly visible in the later models, where both the infected and recovered classes are divided into subclasses (such as, *asymptomatic*), which have different time scales and/or transmission modes. In this review, efforts have been taken to group the epidemiological models of malaria in terms of the complexity of infection processes included in its description, which makes them more realistic. The age-specific distribution of infection due to differential immunity across age is one such case. The assumption is that more realistic models would enhance the understanding of the infection transmission process at the population level, which, in turn, may help in better prediction of intervention strategies. The specific models discussed here are only indicative and not exhaustive. Pure mathematical analysis of the models, even though not so popular among the biologists, is important. They allow clear understanding of the logic of the system behaviour in terms of the relationship among the parameters and variables, which are representative to real biological processes. It will be useful to develop connections between mathematical analysis and their real world implications, since such analyses may help us to understand hitherto unknown scenario, such as the effect of temperature, seasonal forcing, excessive rainfall, correlation between different variables and parameter changes. Among the innumerable statistical models based on malaria incidence data, only a few approaches have been described here. The results of these models are mostly data specific and applicable primarily to the particular data set studied. They are highly useful for prediction in that specific context, but may not work in other places/scenarios. Models that incorporate the essentials of host-parasite-vector interaction, proper clinical population subdivisions for disease transmission and also describe multiple data sets from different ecological regions, promise to be an ideal combination of both approaches. It is now clear that the role of indirect factors such as, social structure, economic status, play an overwhelming role in the transmission and persistence of malaria in a region. It also underlies the failure of several control measures where local heterogeneity was not considered. It is the need of the hour to include factors such as the role of heterogeneity in host population due to social status, local differences in ecology due to poverty, differential effects of disease transmission in populations residing in habitats of different temperature, in the mathematical models. Such a description has the possibility of yielding understanding of malaria transmission for populations with societal differences and climate change. Mathematical models have the ability to address several multiplicative, feedback and nonlinear effects that enhance or suppress the effects of factors such as, exposure, immunity, spatiotemporal heterogeneities, control measures and environment, in order to capture key linkages to the complex transmission dynamics. They can also include stochasticity in different variables and parameters to simulate realistic scenario. This comparative analysis of different mathematical models of malaria would contribute to consolidate our understanding about the evolution of these models, and may also help in developing new models by incorporating features discussed above to improve predictions and deciding realistic control measures.

## Competing interests

The authors declare that they have no competing interests.

## Authors' contributions

SM identified literature sources, carried out the analysis, and wrote the manuscript. RRS contributed in designing the study and to the formatting and writing of the manuscript. SS conceived the idea for the article, designed the study, and contributed to the writing of the manuscript. All authors have read and approved the final manuscript.

## Supplementary Material

Additional file 1 **Description of different mathematical models of malaria**. This file contains eight Tables (S1-S7i,ii), giving description of mathematical expressions and parameters used in different models. Click here for file

Additional file 2 **Description of different immunity functions**. This file contains details of three immunity functions used in Filipe model [44]. Click here for file
