# Supplementary material for: Mathematical models of malaria - a review
Source: Malar J. 2011 Jul 21;10:202. doi: 10.1186/1475-2875-10-202 (PMC3162588; doi:10.1186/1475-2875-10-202)
Supplement: Additional file 1 — Description of different mathematical models of malaria. This file contains eight Tables (S1-S7i,ii), giving description of mathematical expressions and parameters used in different models. [file 1475-2875-10-202-S1.DOC]

**Additional file 1**

**Table S1:** Ngwa-Shu model [1]

| **Model** | **Parameters** |
| --- | --- |
|  | $g_{h}/g_{m}$- birth rate of human/mosquito  $\gamma$*-* rate of loss of immunity  $\nu_{h} /\nu_{m}$-infectious rate from exposed  class for human / mosquito  $\mu'_{1}$/$\mu_{1}$*-* density independent / dependent  death rate of human  $\mu_{d}$*-* disease induced death rate of  human  $\mu'_{2}$/$\mu_{2}$*-* density independent /dependent  death rate of mosquito  $q$*-* acquire immunity rate  $C_{m h}$-infectivity of mosquito  $C_{hm }$- infectivity of infected human (*I_h_*)  $\tilde{C}_{hm}$- infectivity of immune human (*R_h_*)  *a* - biting rate of mosquito on human  *r-*average recovery rate of human from  infectious to susceptible class  $N_{h} /N_{m}$-total number of human/mosquito |

**Table S2:** Chitnis model [2]

| **Model** | **Parameters** |
| --- | --- |
| Mosquito dynamics is same as Ngwa model [Table S1] | $\Lambda$*-* immigration rate of human  All other parameters are same as in Table S1. |

**Table S3:** Yang model [3]

| **Model** | **Parameters** |
| --- | --- |
|  | $g_{h}$- birth rate of human  $\mu_{1}$- natural mortality rate of human  $\mu_{d}$- disease induced mortality rate of human  $\theta$- natural resistance rate against malaria  $h'$- force of infection produced by each infected mosquito  $\pi_{1}$-rate of loss of protective immunity  $\pi_{2}$- rate of loss of partial immunity  $\gamma$- rate of loss of immunological memory  $\nu_{h}$- rate of production of gametocytes  $q$- acquire immunity rate  $\Phi$- rate of oviposition  $\sigma_{1}(T)$-rate of becoming adult from egg  $\sigma_{2}^{-1}(T)$-duration of sporogony in the mosquito  $\mu_{e}(T)$- rate at which eggs becoming nonviable  $\mu_{2}$- natural mortality rate of mosquito  $\mu'$-induced mortality rate of mosquito  $f'$- rate of transmission of susceptible to infectious  mosquito |

**Table S4:** Filipe model [4]

| **Model** | **Parameters** |
| --- | --- |
| $\begin{matrix} \frac{\partial S_{h}}{\partial t}+\frac{\partial S_{h}}{\partial\alpha}=-h(\alpha) S_{h}+\varphi R_{D} I_{h1}+r_{U} I_{h3} \\ \frac{dE_{h}}{dt}+\frac{dE_{h}}{d\alpha}=h(\alpha) S_{h}-\tau_{h}^{-1}E_{h} \\ \frac{dI_{h1}}{dt}+\frac{dI_{h1}}{d\alpha}=\varphi\tau_{h}^{-1} E_{h}+\varphi h(\alpha) I_{h2}-\left(R_{D}+\mu_{d}\right) I_{h1} \\ \frac{dI_{h2}}{dt}+\frac{dI_{h2}}{d\alpha}=(1-\varphi) \tau_{h}^{-1} E_{h}+(1-\varphi)R_{D}I_{h1}-\varphi h(\alpha) I_{h2}-r_{A}I_{h2} \\ \frac{dI_{h3}}{dt}+\frac{dI_{h3}}{d\alpha}=r_{A}I_{h2}-r_{U}I_{h3} \end{matrix}$ With, $R_{D}=f r_{T}+(1-f) r_{D}$ and $h(\alpha)=(EIR) b (1-e^{-\frac{\alpha}{\alpha_{0}}})=(m a I_{m}) b (1-e^{-\frac{\alpha}{\alpha_{0}}})$ | $h(\alpha)$- force of infection experienced by  a person of age  $\alpha$ $\tau_{h}$- latent period of human $\varphi$ -proportion that develop symptomatic  disease $f$ -proportion of symptomatic cases  who receive treatment  $r_{T}$- recovery rate with treatment $r_{D}$ - natural recovery rate without  treatment $r_{A}$ -rate at which infectious become  subpatent $r_{U}$ - rate of clearance of subpatent  infection  $\mu_{d}$-disease induced mortality  $\alpha_{0}$- age at which half the total increase  in exposure is achieved  *EIR*-entomological inoculation rate  *a* - biting rate of mosquito on human  *b-* proportion of bites that produce  infection on humans  *m-* number of female mosquitoes  relative to human |

**Table S5:** Koella-Antia model (resistant strain) [5]

| **Model** | **Parameters** |
| --- | --- |
| $\begin{matrix} \frac{dS_{h}}{dt}=g_{h}-\mu_{1} S_{h}-(h_{s}+h_{r})S_{h}+\gamma R_{h} \\ \frac{dI_{h1}}{dt}=f h_{s} S_{h}-(r_{st}+\mu_{1}) I_{h1} \\ \frac{dI_{h2}}{dt}=(1-f) h_{s} S_{h}-\left(r_{su}+\mu_{1}\right) I_{h2} \\ \frac{dI_{h3}}{dt}=h_{r} S_{h}-\left(r_{r}+\mu_{1}\right) I_{h3} \\ \frac{dR_{h}}{dt}=r_{st} I_{h1}+r_{su}I_{h2}+r_{r} I_{h3}-(\gamma+\mu_{1})R_{h} \end{matrix}$ With $\begin{matrix} h_{s}=m b_{s} a^{2} e^{-\mu_{2}\tau_{m}}\frac{I_{hs}}{\mu_{2}+a (b_{s}I_{hs}+b_{r}I_{h3})} \\ h_{r}=m b_{r} a^{2} e^{-\mu_{2}\tau_{m}}\frac{I_{h3}}{\mu_{2}+a (b_{s}I_{hs}+b_{r}I_{h3})} \\ I_{hs}=I_{h1}+I_{h2} \end{matrix}$ | $g_{h}$- birth rate of human  $\mu_{1}$- natural mortality rate of human  $h_{s}$*-* inoculation rate for anti malarial sensitive  $h_{r}$*-*inoculation rate for drug resistant  $r_{st}$*-* recovery rate from infection for treated  $r_{su}$*-* recovery rate from infection for untreated  $r_{r}$*-* recovery rate from infection for resistant strain  $\gamma$-rate of loss of immunity  $f$- percentage treated  $b_{s}$-proportion of bites that produces sensitive strain on human  $b_{r}$- proportion of bites that produces resistant strain on human  *a* - biting rate of mosquito on human  *m-* number of female mosquitoes relative  to human  $\mu_{2}$-mosquito death rate  $\tau_{m}$- latent period of mosquito |

**Table S6:** Parham-Michael model [6]

| **Model** | **Parameters** |
| --- | --- |
| $\begin{matrix} \frac{dS_{h}}{dt}=-a(T) b I_{m} S_{h}/N_{h} \\ \\ \frac{dI_{h}}{dt}=a(T) b I_{m}(t-\tau_{h}) S_{h}(t-\tau_{h})/N_{h}-r I_{h} \\ \\ \frac{dS_{m}}{dt}=\lambda(R,T)-a(T) c I_{h} S_{m}/N_{h}-\mu_{2}(T) S_{m} \\ \\ \frac{dE_{m}}{dt}=a(T) c I_{h} S_{m}/N_{h}-\mu_{2}(T)E_{m} \\ -a(T) c I_{h}(t-\tau_{m}(T)) S_{m}(t-\tau_{m}(T)) l_{m}(T)/N_{h} \\ \\ \frac{dI_{m}}{dt}=a(T) c I_{h}(t-\tau_{m}(T)) S_{m}(t-\tau_{m}(T)) l_{m}(T)/N_{h} \\ -\mu_{2}(T) I_{m} \end{matrix}$ With $N_{h}=S_{h}+I_{h}+R_{h}$ and $\lambda(R,T)=BP_{E}(R)P_{L}(R)P_{L}(T)P_{P}(R)/(\tau_{E}+\tau_{L}(T)+\tau_{P})$ | *R*-Rainfall  *T*- Temperature  λ (*R,T*)- Adult mosquito birth rate per day  $a(T)$- biting rate of mosquito on  human  *b-* proportion of bites that produce  infection on humans  *c-* proportion of bites that produce  infection on mosquitoes  $\mu_{2}(T)$-mosquito death rate  *r-*average recovery rate of human  from infectious to susceptible  class  $\tau_{m}(T)$- latent period of mosquito  $\tau_{h}(T)$-latent period of human  $l_{m}(T)$-Survival probability of infected  mosquitoes over the incubation  period of the parasite  *P_E_, P_L_, P_P_*- daily survival probabilities of eggs, larvae and pupae  *τ_E_, τ_L_, τ_P_*-duration of egg, larvae and pupae stages respectively |

**Table S7(i):** Torres-Sorando-Rodriguez model (Migration) [7]

| **Model** | **Parameters** |
| --- | --- |
| $\begin{matrix} \frac{dn_{i}}{dt}=\sum_{j\neq i} e_{ji}n_{j}-\sum_{j\neq i} e_{ij}n_{i} \\ \frac{dI_{hi}}{dt}=a b m I_{mi}\left[n_{i}-I_{hi}\right]-r I_{hi}+\sum_{j\neq i} e_{ji}I_{hj}-\sum_{j\neq i} e_{ij}I_{hi} \\ \frac{dI_{mi}}{dt}=a c I_{hi}\left[\frac{M}{A}-I_{mi}\right]-\mu_{2} I_{mi} \end{matrix}$ | *a* - biting rate of mosquito on human  *b-* proportion of bites that produce  infection on humans  *c-* proportion of bites that produce  infection on mosquitoes  $\mu_{2}$-mosquito death rate  $\mu_{1}$-human death rate  *r-*average recovery rate of human from  infectious to susceptible class  $M$-total mosquito density  *A-*number of fragmentation of the total area  *e_ij_* – migration intensity from i^th^ patch to j^th^ patch |

**Table S7(ii):** Torres-Sorando-Rodriguez model (Visitation) [7]

| **Model** | **Parameters** |
| --- | --- |
|  | *T_ij_*- visitation time  All other parameters are same as in Table S7(i). |

**References:**

1. Ngwa GA, Shu WS: **A mathematical model for endemic malaria with variable human and mosquito populations**. *Math Comput Model* 2000, **32**:747-763.
2. Chitnis N, Cushing JM, Hyman JM: **Bifurcation analysis of a mathematical model for malaria transmission**. *SIAM J Appl Math* 2006, **67**:24-45.
3. Yang HM: **Malaria transmission model for different levels of acquired immunity and temperature-dependent parameters (vector)**. *Revista de Saúde Pública* 2000, **34**:223-231.
4. Filipe JAN, Riley EM, Darkeley CJ, Sutherland CJ, Ghani AC: **Determination of the processes driving the acquisition of immunity to malaria using a mathematical transmission model**. *PLoS Comput Biol* 2007, **3**:2569-2579.
5. Koella JC, Antia R: **Epidemiological models for the spread of anti-malarial resistance**. *Malar J* 2003, **2**:3.
6. Parham PE, Michael E: **Modeling the effects of weather and climate change on malaria transmission,** *Environ Health* *Perspect* 2010, **118**:620-626.
7. Torres-Sorando L, Rodriguez DJ: **Models of spatio-temporal dynamics in malaria**. *Ecol Model* 1997, **104**:231-240.
