# Supplementary material for: Mathematical models of malaria - a review
Source: Malar J. 2011 Jul 21;10:202. doi: 10.1186/1475-2875-10-202 (PMC3162588; doi:10.1186/1475-2875-10-202)
Supplement: Additional file 2 — Description of different immunity functions. This file contains details of three immunity functions used in Filipe model [44]. [file 1475-2875-10-202-S2.DOC]

**Additional file 2**

**Immunity functions**

**Immunity function 1 (IF1):** This function has a value at birth (maternal immunity) conferred vertically with half life, $d_{m}$, and accumulates due to exposure (clinical immunity) with half life $d_{s}$.

The dynamics of immunity function 1 can be described by the partial differential equation:

(B1)

where,$I_{s} (t, 0)$is the immunity at birth (~30, estimated in this paper). The response of immunity function-1 i.e. susceptibility ($\varphi$) is assumed to vary by the following way:

$\varphi[I_{s}(\alpha)]=\frac{1}{1+\left(\frac{I_{s}(\alpha)}{I_{s}(0)}\right)^{2}}.$ (B2)

**Immunity function 2 (IF2):** This function is responsible for clearance of detectable parasites. The recovery rate from detectable parasite ($r_{A}$) is assumed to be a saturating increasing function of the immunity level$I_{A}(t, \alpha)$:

$r_{A}\left[I_{A}(\alpha)\right]=r_{0} \left[1+(w_{A}-1)\frac{\left(\frac{I_{A}}{H_{A}}\right)^{2}}{1+\left(\frac{I_{A}}{H_{A}}\right)^{2}}\right],$ (B3)

where $r_{0}$is the baseline rate of recovery (~1/180 day^-1^), $w_{A}$denotes maximum amplification of baseline recovery rate (30, estimated in this paper), $H_{A}$ is the level of parasite immunity at half saturation (800, estimated in this paper).

Here, $I_{A}(t, \alpha)$can be found from the following sets of equations of ‘parasite immunity level during latent period ($J_{A}$)’ and ‘immunity level ($I_{A}(\alpha)$)’:

$\begin{matrix} \frac{\partial J_{A}}{\partial t}+\frac{\partial J_{A}}{\partial\alpha}=h(\alpha)-\frac{J_{A}}{d_{l}}, \\ \frac{\partial I_{A}}{\partial t}+\frac{\partial I_{A}}{\partial\alpha}=\frac{J_{A}}{d_{l}}-\frac{I_{A}}{d_{A}}. \end{matrix}$ (B4)

Here, $h(\alpha)=h (1-e^{-\frac{\alpha}{\alpha_{m}}})$is the age dependent force of infection, $\alpha_{m}$is the maximum age of human, $d_{l}$(~10 year) is the latent period in development of parasite immunity and $d_{A}$(~20 year) is the half life of parasite immunity.

**Immunity function 3 (IF3):** Clearance of sub-patent infections occurs due to immunity function 3. The rate of clearance ($r_{U}$), which depends on the force of infection and baseline average duration of sub-patent infections $d_{U}$(~180 year), is described by the relation:

(B5)
